# Leveraging Micro-Stories to Build Engagement, Inclusion, and Neural Networking in Immunology Education

**DOI:** 10.3389/fimmu.2019.02682

**Published:** 2019-11-28

**Authors:** Kara Lukin

**Affiliations:** ^1^Department of General Education, Western Governors University, Salt Lake City, UT, United States; ^2^Department of Integrative Biology, University of Colorado Denver, Denver, CO, United States

**Keywords:** immunology education, storytelling, neural networking, retention, diversity, inclusion

## Abstract

Storytelling is a highly effective strategy for delivering course content. It can provide real-world contexts and the relevance students desire. Through personal connections to the narrative details, anecdotes facilitate the incorporation of content into pre-existing knowledge and neural networks that enhances retention. In addition, stories can honor students' diverse backgrounds, which builds a sense of belonging and community. In turn, these aspects can drive intrinsic motivation to learn and increase students' alertness in class and overall engagement in the course. Despite the educational power of stories, there often is not enough time to integrate them into curricula. To address this dilemma, faculty can condense stories into micro-stories that require relatively minimal class time. Many aspects of stories that enhance learning can be leveraged in just a few sentences by focusing on narrative details that engage a variety of cognitive and emotional processes. In particular, the inclusion of multiple sensory descriptions and small details, like locations and names, can provide sufficient context to maintain the value stories provide. Micro-stories can function independently or extend a single theme throughout a course. Presented in this Perspective are examples of micro-stories for concepts in immunology and strategies for developing them. Proposals are made for leveraging micro-stories to enhance student engagement and course community, content retention and retrieval, and satisfaction with immunology courses of all sizes and levels.

## Introduction

On a chilly Fall afternoon, a student asked for class to be composed solely of stories about immunology. His classmates laughed and agreed they would not return if there were no stories the rest of the term. Although in jest, the comments underscore that many students find stories about science more enjoyable and easier to understand than dense textbooks and lecturing (1–3). Fortunately, leveraging stories to convey science content also can enhance academic outcomes (3–6).

Stories are one of the frameworks human beings use to process experiences and understand the world (7). They provide a familiar paradigm for learning complex and interwoven material while eliciting strong engagement from many students. The engagement is often driven both through a natural desire to uncover a story's conclusion and varied affective (emotional) motivations (8, 9). Robust social-emotional associations with course material, educators, and the course community can lead to increases in attentiveness in class and personal motivation, which can promote deeper levels of learning synergistically (8–10). In turn, deeper learning enhances the formation of memory and recall of information.

Memory can be viewed as hubs of interconnected records of one's experiences and the information that one has learned. These networks provide a scaffold to which new information can be incorporated by association. The more associations that connect new information to existing neural networks, the more easily the information can be recalled (11). In addition, the more associations that are activated when retrieving information, the more strongly the information will be maintained (12). Thus, the contextual details of stories can enhance retention of immunology concepts by connecting the concepts to memory networks containing information beyond science. Strong sensory descriptors can stimulate mental imagery and reactivate the sensory and motor cortices which initially processed the sensations (11, 13). In both cases, the associations between content and real or visualized experiences can increase the ability to retain and recall specific information (14–16). Cuevas and Dawson demonstrated that university students who invoked visual imagery while hearing statements recalled two-fold more than students in the auditory-only cohorts in the short term, regardless of learning style preferences (81.9 and 40.2% correct answers, respectfully).

Despite storytelling's power as an educational tool, faculty face a conundrum regarding its implementation. How can one balance the use of this effective strategy beloved by many students with the time needed to cover necessary content? Even with calls to reduce content to incorporate social learning strategies and relevant applications of science, it is difficult to pare down the material to create time to leverage storytelling (17, 18). Employing micro-stories can address this issue. Micro-stories are terse narratives that focus on specific sensory and contextual details to harness the affective and cognitive benefits of storytelling in minimal amounts of time.

This Perspective shares strategies to develop and infuse course materials and class time with micro-stories that can increase student learning outcomes by succinctly (1) invoking associations among content and students' existing neural networks, (2) enhancing students' intrinsic motivation by strengthening course communities, and (3) reinforcing content retention through distributed recall. Overall, the goal is to empower educators to incorporate new strategies or extend current ones that enhance student performance and enjoyment of immunology.

## Developing Micro-Stories to Enhance Learning and Memory

When constructing micro-stories, it is helpful to incorporate some of the features that make case studies appealing to students: characters with whom students feel empathy, an interest-arousing focus like social conflict, drama or adventure, and personal relevance (19, 20). Providing first names and using female and male protagonists fosters affinity for the characters. Details about the setting can help transport students into micro-stories and stimulate imagery (perceiving in “the mind's eye”). These features can be specific geographic locations, like New York City, or general ones, like the campus' library or a room of a home. Adventure can be introduced by setting the micro-story in the midst of an exciting activity, distant city or foreign country. Social issues at the community, national, and global levels, like requirements for vaccination, can be extremely engaging and need an inclusive approach (21). Elements of micro-stories that relate to the five senses and engender empathy are especially critical because they facilitate students' engagement with the narratives and build connections to students' personal experiences. This can link the course material to varied neural networks which can enhance retention and retrieval of the immunology concepts, even if a story's details don't exemplify an immunologic concept. The following example conjures a familiar setting for many students with relatable visual, auditory, and tactile components. Some students will empathize with Cody being a weaker student and some with Yolanda as the stronger one. “The campus center was noisier and more packed than usual when Cody arrived to study with Yolanda. He was grateful to get out of the rain and for Yolanda's help preparing for their immunology midterm. However, Cody was nervous about being in a crowded area because he has chronic granulomatous disease (CGD). Although his case is relatively mild, he is susceptible to respiratory infections and worries about pneumonia due to antibiotic resistant *S. aureus*.” After disclosing the aliment, the instructor can interrupt the story so students can hypothesize about Cody's disease and symptoms.

The next example directly ties the micro-story to a role of IgE. Faculty could spend just 60 additional seconds capturing students' attention and imagination with a micro-story like: “Olivia moved to Alaska to explore its wilderness during time off. After a day of fly-fishing, she stuffed a salmon she caught with garlic cloves and sprinkled it with salt and pepper. It smelled delicious as it blackened rapidly over the campfire. But, the fish didn't cook completely. Unfortunately, Olivia contracted a tapeworm. Hopefully, she will produce IgE antibodies specific for the parasite.” The details quickly create adventure and, hopefully, empathy for Olivia. Although most students probably have not traveled to Alaska, the details make the micro-story accessible. Students may have experience camping or be able to imagine the tastes and smells of the fish. Many students may have had an experience with raw or undercooked meat, seafood, or fish. The goal is to aid students' recall of the concept by coupling it to established memories. In addition, asking students to reflect for a moment on a related personal experience constructs bridges between micro-stories and their memory networks.

**Figure 1:**
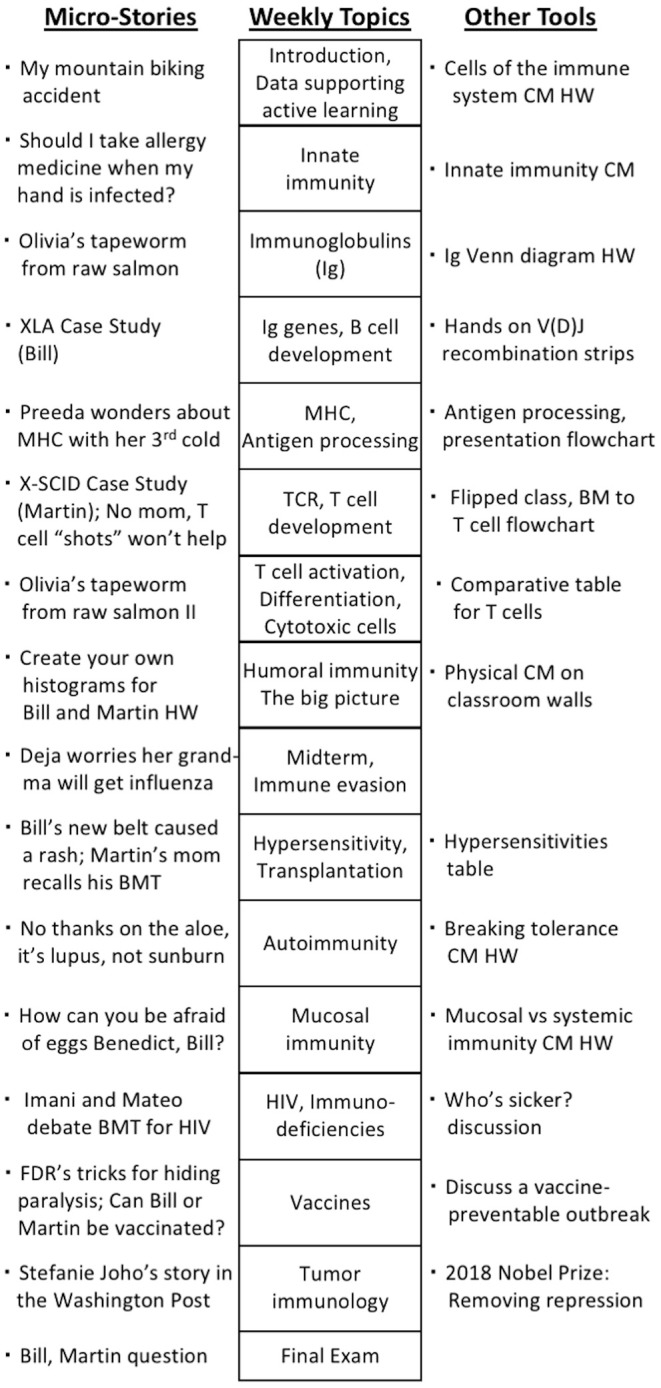
Micro-story topics and other learning tools used in a 16-week immunology course. The topics covered each week are indicated in the center. The themes of micro-stories told with each topic are on the left. Other learning tools and homework assignments are indicated on the right. Several of the homework assignments are started in class. The case studies are discussed in full. The micro-stories and examples of their implementation are provided in Supplementary Table 1. BMT, bone marrow transplant; CM, concept map; FDR, franklin delano roosevelt; HW, homework; Ig, immunoglobulin.

If writing micro-stories seems daunting, they can be created by condensing stories from a variety of sources. Newspaper articles (for example, about anti-PD-1 cancer treatments) and single-paragraph highlights in *Nature* and *Science* (for example, concerning patients who appear HIV-free following stem cell therapy) demonstrate direct application of concepts of immunology (22, 23). Micro-stories can also be distilled from historical anecdotes. For example, Charles Richet and Paul Portier's attempts to develop antivenom to the stings of Portuguese man o' war lend themselves to describing the smell and taste of salty sea air, the swaying of the vessels and/or the pain of the stings. Such a micro-story could conjure adventure and empathy, activate multiple sensory networks, and create personal connections, even if students recall an insect sting instead of a hydrozoan sting. Ultimately, there are countless micro-stories faculty could develop to meet the needs of their specific student populations (Figure 1 and Supplementary Table 1).

## Micro-Stories Cultivate Community Via Shared Experiences

A strong sense of course community increases student engagement and performance (24, 25). Robust instructor immediacy (students' feelings of closeness to their educator) considerably strengthens the course community (26). When instructor immediacy is high, students' attention, motivation, effort, and willingness to ask questions increase (27, 28). In turn, the perception of having learned, actual learning, and student performance all increase as well (29, 30). Sharing personal experiences, humor, and one's mistakes demonstrates to students that educators are human and enhances instructor immediacy (31). Even in large-enrollment courses, the safety such behaviors instill can encourage students to forge relationships with each other and seek help outside of class (27).

When discussing barriers, innate responses, or integrated immune responses, an educator could share about an accident or infection: “My first summer of graduate school, I went mountain biking. The woods smelled like the pine air fresheners used in cars. I took a turn too quickly, hit a tree and broke a few fingers. The cuts burned and were full of debris. What do you think got into my hand? How do you think my immune system responded?” The sensory details can assist students in relating to the anecdote even if they haven't been mountain biking. Colds, food poisoning, and hypersensitivities are other compelling topics for micro-stories because they are experiences about which students probably can commiserate with each other and faculty.

A sense of belonging among students can be fostered when students share their own, relevant micro-stories. This provides opportunities for weaker students to contribute to class as experts because their experiences exemplify immunology. It is critical to emphasize that it is ok to share; however, sharing is not requested or required, nor will it impact grades. Students should not feel pressured to share personal information. In the author's courses, students have been eager to discuss being resuscitated after a reaction to peanuts, battling vitiligo, being on the autism spectrum and more common topics.

## Diversity in Micro-Stories Builds Inclusivity

With the expanding diversity of student populations, cultivating a sense of inclusion of all students in course communities is essential. Thus, micro-stories need to represent varied ethnic, racial, geographic, gender, and economic backgrounds as well as first-generation students. This recognition fosters a sense of belonging in the class and institution, which can increase interactions with faculty, engagement, motivation, development of cognitive skills, retention at the institution, and overall outcomes (25, 32, 33).

A simple way to incorporate diversity in micro-stories is using global settings and culturally diverse, but not stereotypical, names. Names with multiple origins increase self-identifying with them. Amana and Tam are East African and Middle Eastern names, with Tam also being Pan-Asian and Scottish. Lina and Kai are equally diverse and, with Isabella, Jasmine, Martin, Micah, and Tyler, span other populations of the United States. Diversity can also be introduced with examples that are relevant to student demographics like levels of MHC diversity (34). Micro-stories that compare relationships among the old friends hypothesis, allergy, autoimmunity, and genetic predispositions could use examples from rural vs. urban areas and similar incidences of type 1 diabetes in youth in Algeria and the United Kingdom (35). In addition, a micro-story about vaccination could focus on the early use of variolation in India, Asia, and/or the Ottoman Empire instead of Edward Jenner's overshadowing experiment.

Forms of diversity that are less obvious in the classroom also require consideration. For example, a plot might involve Kiara explaining to her wife, Beth, why she can't be a bone marrow donor for their nephew. Zayd might share his excitement about his summer research position and his concerns about finding another job in the Fall. With all micro-stories, it is very important to highlight positive qualities and avoid negative associations to prevent stereotype threat. Stereotype threat is a fear of conforming to a negative stereotype about one's social group. Anxiety from stereotype threat prevents students from focusing on course work and reduces academic performance (36).

## Never-Ending Stories Drive Retrieval and Retention

Case studies are excellent vehicles for increasing critical thinking skills, comprehension, and retention; however, they can take time to work through. By combining a case study with a series of micro-stories about the case's protagonist, the tools synergize. A short case study can be the anchor to which information in micro-stories is related throughout a course. If students know the protagonist and his or her situation well, it requires minimal class time to review the immunologic condition and ask students about the impact of a new concept. As students continually revisit the protagonist's situation, they solidify concepts via increased networking and distributed retrieval (repeatedly recalling information at intervals, Figure 1 and Supplementary Table 1). When learning from science texts, including biology, 84% of students using retrieval practice performed better on short answer questions than students using elaborative studying with concept maps [means of proportion correct were 0.73 and 0.54, respectively (37)].

The strategy used by the author introduces two short case studies early in the term about agammaglobulinemia (XLA) and X-linked severe combined immunodeficiency (SCID); however, one is sufficient (38). A variety of free, peer-reviewed immunology cases is available at the National Center for Case Study Teaching in Science (39). For brevity, focus here is on the XLA patient Bill. The case itself addresses the roles of antibodies in immune responses, B cell development, signaling processes downstream of the B cell receptor, and flow cytometry. Students learn Bill's name and condition by discussing these topics. By extending Bill's narrative through micro-stories, the immunologic principles above can be recalled frequently. Similarly, new concepts related to Bill's health and immunodeficiency can be incorporated through the term. For example, a micro-story about Bill's transition to college bolsters discussions of vaccine types (Supplementary Table 1). Bill's skin reaction to a belt his girlfriend gave him can enliven the exploration of the types of hypersensitivities. Bill's frustration at not being able to donate blood for a campus drive can remind students about the half-lives of immunoglobulins and passive immunity.

Micro-stories related to a single protagonist facilitate interleaving of course material. Interleaving is a strategy of reviewing related, but different concepts instead of focusing on a single concept at a time (40). The approach presses the brain to differentiate concepts and focus on details instead of being lulled into a false sense of knowing the information. With the example above, students' familiarity with Bill's situation allows faculty to re-visit multiple principles of immunology in quick succession. The power of this never-ending story is exemplified by a student's email months after his immunology course concluded (Jorge Dominguez, personal communication April 17, 2019):

“Dear Dr. Katja and Lukin

I saw this in the news and instantly was reminded of your case studies with Bill and Martin … https://www.google.com/amp/s/amp.cnn.com/cnn/2019/04/17/health/bubble-boy-disease-cure-study/index.html

Jorge”

## Discussion

Immunology is a continually expanding field in which complex and interwoven concepts are abundant. In part, this results in many students struggling with and not enjoying the coursework. Instructional storytelling is a powerful education tool that students find pleasurable and engaging (3, 5). However, it can consume class time needed to cover content. The “six-word story” genre attributed to Ernst Hemingway demonstrates that stories need not be lengthy to capture attention (For sale: Baby shoes. Never worn). This paper suggests that terse micro-stories can promote engagement and associative learning. By constricting narratives to key contextual details, micro-stories can be told in <2 min or presented in a few sentences. Consequently, these anecdotes can be incorporated into class discussions and assessment items and interwoven with other learning tools frequently (Figure 1).

Micro-stories have the potential to connect principles of immunology to memories of common sensory perceptions, personal experiences, and newly created imagery of scenarios. Linking the principles to memory networks and imagery can facilitate learning and subsequent retrieval (14, 16). As recollections focus the networks to which the information is linked, memory of the material is more rapid (12). By employing micro-stories about themselves and to recognize diversity in the class community, educators can enhance instructor immediacy and students' perceptions of belonging, which can augment the development of cognitive skills and overall success (25, 27, 33). Moreover, the core aspects of micro-stories align with andragogy (the teaching of adult learners). Thus, micro-stories can help faculty support the needs of this growing population across all levels of post-secondary education in the United States (32, 41).

The examples presented here are based on experiences with diverse, urban student populations. Since student demographics vary greatly among institutions, the suggestions about unifying factors in the micro-stories will need to be adapted to each student population. The flexibility of the contextual details facilitates this. While micro-stories can be effective for classes of any size enrollment, collecting data about students' interests to tailor micro-stories may be challenging with high-enrollment courses.

To reduce barriers to adopting micro-stories, faculty are encouraged to integrate the tool incrementally. Educators can focus first on a few key concepts with which students often struggle. If sharing personal experiences is uncomfortable, avoid them. Students may perceive them as false intimacy, false intimacy, which could produce negative feelings. Enhancing available resources, like examples in end of chapter practice questions, can expediate the development of micro-stories. Also, the tool is not restricted to in-class discussion. Micro-stories can be incorporated into practice and assessment questions to elevate students' focus and enthusiasm for the tasks. Similarly, the development of micro-stories as homework or group work allows students to express their creativity (Supplementary Table 1). High-quality submissions can be leveraged in subsequent courses. Despite their potential benefits, it is likely that not all students will enjoy micro-stories. This can be addressed when educators explain their teaching styles each term.

This Perspective suggests that the incorporation of micro-stories into immunology curricula can enhance student engagement, motivation, satisfaction, and academic outcomes. However, a significant limitation is the lack of assessment of the tool. Future studies are required to evaluate the impact of micro-stories on the affective and cognitive dimensions of student performance. It will be important to assess these independently and to determine their interdependence. In addition, dissecting the impacts of micro-stories on different types of course assessments will be valuable. Together, this analysis will inform educators on how to leverage the tool to support students most effectively.

## Author Contributions

The ideas in this Perspective article were developed by the author. The author is the sole contributor to this manuscript and approves the manuscript.

### Conflict of Interest

The author declares that the research was conducted in the absence of any commercial or financial relationships that could be construed as a potential conflict of interest.
