# Supplementary material for: Leveraging Micro-Stories to Build Engagement, Inclusion, and Neural Networking in Immunology Education
Source: Front Immunol. 2019 Nov 28;10:2682. doi: 10.3389/fimmu.2019.02682 (PMC6893969; doi:10.3389/fimmu.2019.02682)
Supplement: Supplementary file 1 [file Table_1.docx]

Supplementary Material

**Supplementary Table 1.** The information in Figure 1 concerning the topics of micro-stories used to support each weekly topic for a sixteen-week immunology course is expanded. Specific micro-stories, case studies, homework and exam questions indicated in Figure 1’s left column are provided with explanations of how some of the items are used in class.

| **Micro-Story Title** | **Class Topics** | **Micro-Story and Possibilities for Implementation** |
| --- | --- | --- |
| My mountain biking accident | Introduction to immunology | My first summer of graduate school, I went mountain biking. The woods smelled like the pine air fresheners used in cars. I took a turn too quickly, hit a tree and broke a few fingers. The cuts burned and were full of debris.  The class is asked: What do you think got into my hand? How do you think my immune system responded?  Note: An educator could share details of any personal story that allows the discussion of breaching immune barriers. |
| Should I take my allergy medicine? | Innate immunity | On a warm summer afternoon, I reached out to pull a few weeds that were wrapping around the mint plant in my front yard. I was shocked to be stung by a bee. My ragweed allergies were in full swing, but luckily I am not allergic to bee venom. Unfortunately, by the evening, my hand was so swollen I could not close it to make a fist. My biggest dilemma that night was whether or not to take my allergy medication which blocked mast cell degranulation. I was sure I had a bacterial infection from the bee sting so I didn’t want to hinder an immune response, but I didn’t want to spend the night sneezing and blowing my nose either.  Students are asked why I was struggling so much with this decision as a way to review the role of mast cells in innate immunity. |
| Olivia’s tapeworm | Immuno-  globulins | Olivia moved to Alaska to explore its wilderness during her time off. After a day of fly-fishing, she stuffed a salmon she caught with garlic cloves and sprinkled it with salt and pepper. It smelled delicious as it blackened rapidly over the campfire. But, the fish didn’t cook completely. Unfortunately, Olivia contracted a tapeworm. Hopefully, she will produce IgE antibodies specific for the parasite. |
| XLA Case Study (Bill) | B cell development | We work through a case study about agammaglobulinemia (XLA) found in *Case Studies in Immunology: A Clinical Companion* (Geha 2016). The protagonist’s name is Bill. |
| Preeda wonders about MHC with her third cold of the season | MHC, Antigen processing | Preeda felt lucky that her mother was bringing over a large batch of her favorite coconut soup and unlucky that the “special delivery” was because she had caught another cold. It was the third cold this winter. It was starting to get annoying. At least it was the weekend so she didn’t have to drag herself to class and could rest until her mother arrived. As she thought about how the hot chilis in the soup would help clear her nose up for at least a little while, Preeda started wondering about the MHC molecules that she was learning about in immunology class and how she processes antigens. Was it possible that something related to this could be making her more susceptible to catching colds?  The question is opened up to the class for discussion or for a written “spot check” to evaluate students’ progress with the material. |
| X-SCID Case Study (Martin) | T cell development | We work through a case study about X-linked severe combined immunodeficiency (X-SCID) found in *Case Studies in Immunology: A Clinical Companion* (Geha 2016). The protagonist’s name is Martin. |
| No mom, T cell “shots” won’t help | T cell development | Tomas is so excited to hike the Inca Trail all the way to Machu Picchu in the Andes Mountains over Spring break. While he is thinking about the lush emerald green trees and wisps of clouds in the Peruvian mountains, his mother is thinking about Yellow Fever and Zika virus that she has heard about in the news. She thinks Tomas will catch a fatal disease while traveling. She read on the internet that T cells are super warriors of the immune system. So, she insists Tomas get an infusion of T cells before the trip. Explain at least two reasons Tomas’ mother’s idea is flawed.  This question is provided for students to discuss in pairs during class or as an exam question to target concepts including self-MHC restriction, TCR specificity for specific antigens and memory. Students often discuss activation of NK cells as a concern. |
| Olivia’s tapeworm, Part II | Cytotoxic cells | Remember our friend Olivia who loves to explore the Alaskan wilderness and didn’t cook the salmon she caught completely?  Students are asked the following questions for review: What happened? Which immunoglobulin were we hoping she would generate to help her eliminate the parasite she contracted? Yes, IgE. Mast cells have receptors for the Fc portion of IgE. Which part of the antibody is the Fc portion? Will this part of Olivia’s IgE proteins be highly variable or constant? Why is it possible for mast cells to have receptors for the Fc portion of IgE, but not the antigen binding portion of the immunoglobulin? Once mast cells have bound IgE by the Fc receptors, how many types of antigens can the mast cell detect?  After this review of Olivia’s adventure, antibody structure and mast cell sensitivity, the roles of mast cells and eosinophils in the elimination of parasites is explained. |
| Create histograms for Bill and Martin homework | The big picture | The following homework assignment is given to students with four histograms (histograms A and C only have negative populations, B has both negative and positive populations and D only has a positive population).  Based on the diseases that Bill and Martin have, complete the following items for the histograms provided.   - 1. Label the X-axis and Y-axis appropriately with fluorescence intensity or cell number.   2. Graphs A and B are histograms for two different proteins.   Label the X-axes so that each histogram could represent samples of white blood cells found in Bill’s lymph nodes.   - 1. Graphs C and D are histograms for two other proteins.   Label the X-axes so that both histograms could represent samples of white blood cells found in Martin’s spleen.   - 1. Explain your choice of marker (protein) for each graph and why the data for each graph aligns with the person’s disease. |
| Deja worries her grandmother will get influenza | Immune evasion | Deja worries about her grandmother because she dotes on all her young grandchildren at family dinners every Sunday evening. Deja knows the elderly have weakened immune responses and the younger children are exposed to so many pathogens at pre-school and daycare. Her little cousins often have runny noses or coughs.  Deja has the same conversation with her grandmother each Fall about getting the flu shot. Although her grandmother doesn’t like needles and she resists, Deja tries to persuade her to get the vaccine by explaining how the influenza virus evades the immune system. She explains that the virus develops many mutations in the genes that encode the key viral entry and exit proteins so the protection generated from the previous year’s vaccine is not as effective against this year’s virus (antigenic drift). Deja also describes that sometimes the changes are even greater. She draws out for her grandmother an example of what happens when the virus changes subtypes of the genes, like from H1 to H3, that they have discussed previously. When this happens, the immune memory from the previous year is not effective against the current year’s virus (antigenic shift).  In the end, her grandmother agrees we all need a different vaccine each year to help the immune system recognize the proteins the virus is using each year. Deja is relieved that she promises to get one on Monday and she will invite her neighbor to go with her. |
| Bill’s new belt caused a rash; Martin’s bone marrow transplant | Hyper-sensitivity, Trans-plantation | Our friend Bill is talking with his roommate, Antwan, about the belt his girlfriend gave him for his nineteenth birthday. Bill explains that he loves the belt. It is exactly what he wanted - a black leather belt with a silvery buckle. The problem is that he developed a rash near his belly button. The nurse at Student Health Services said it was contact dermatitis caused by a reaction to the nickel plating on the belt buckle. Bill is worried that his girlfriend is going to be annoyed by one more health issue and feel she wasted her money. But, Antwan encourages him that honesty is the best policy and suggests it might be possible to return or exchange the belt.  We return to the case study concerning Bill and discuss the details of his treatment. Students are asked which types of hypersensitivities Bill could develop. The potential timelines for the reaction to nickel are discussed as well as if it is likely that the belt is returnable. |
| No thanks on the aloe, it’s lupus, not sunburn | Auto-immunity | It can get a little annoying, but Keon knows people mean well when they offer him all sorts of moisturizer or sunscreen because of the redness on his nose and cheeks. The flowery smelling ones are the worst, he feels. When the rashes first started in college, he was embarrassed. He felt like he had a never-ending case of acne. Over the years, Keon has gotten used to it and is grateful the rashes are his main symptom [an educator could pause here for student predictions about the disease]. Keon explains to his friends that the rash is due to lupus, an autoimmune disease, not windburn from skiing or too much sun exposure. Often, he lets his friends know that he uses a special anti-inflammatory cream for the rash each day so they don’t continue to offer suggestions or their favorite body and hand lotions. |
| How can you be afraid of eggs Benedict, Bill? | Mucosal immunity | Our friend Bill from our case study is having breakfast out with some friends. The restaurant is hopping and the smell of rich coffee permeates the air. Forty Winks is known for their many delicious varieties of eggs Benedict. Marcella teases Bill about his order: “Are you carbo loading for a race or something with those pancakes? Aren’t you going to try one of the eggs Benedict?” Bill replies that eggs Benedict make him nervous because they are not fully cooked so they could be contaminated with live *Salmonella* bacteria. “Oh, come on, don’t be a germaphobe. The eggs might be a little runny, but they are so delicious, especially the ones with spinach and cream sauce,” Marcella pushes. Bill holds his ground and explains that he has to be careful about what he eats because he has XLA.  The class is asked what causes XLA, Bill’s treatment and why Bill might be concerned about eating eggs that could be contaminated with *Salmonella*. This sets up our discussion of the importance of antibodies in the mucosa for clearing pathogens, especially viruses and intracellular bacteria like *Salmonella,* prior to entering the body’s tissues and our cells. |
| Imani and Mateo debate BMT for HIV treatment | HIV, Immuno-  deficiencies | Imani and Mateo are in the same learning group in their immunology course. Most weekends they meet to go over learning objectives and study. Because of their work schedules, they are meeting over the phone this Saturday evening. Imani is glad to avoid trekking out in the snow and to be staying warm at home. Mateo enthusiastically explains the details of an article he saw on Twitter about an HIV+ person who received a bone marrow transplant due to leukemia. The donor had two mutated alleles of CCR5. The recipient was able to stop taking anti-retroviral drugs and seems to be HIV-free. Mateo suggests they don’t have to worry about their homework assignment to design a vaccine for HIV because now there is a cure for HIV infection. Imani interrupts to remind him that finding donors who are bone marrow matches is hard enough, let alone donors who are CCR5-deficient. She points out that bone marrow transplants are expensive, risky and, sometimes, fatal. Imani also reminds Mateo that CCR5-deficiency seems to be limited to a small percentage of people of European decent. Both Imani and Mateo start conjecturing about how advancements in technology might make a CCR5-related HIV treatment a reality someday (Warren 2019). |
| FDR’s tricks for hiding paralysis; Can Bill or Martin be vaccinated? | Vaccines | Close your eyes and image a time when the number of televisions in the United States is in the thousands. It is the 1930’s. People obtain their news from the radio and printed newspapers. At the age of 39, Franklin D. Roosevelt, the future 32^nd^ president, contracted polio which left him paralyzed from the waist down. To assure the public didn’t realize how significant the paralysis was, Mr. Roosevelt and his staff made sure he was never photographed in ways that showed his disability. They orchestrated his arrivals and departures from events so the audience would not see him struggle. For example, ramps were created so his car would be level with any stage. He would not have to climb stairs or walk more than a short distance (holding an aide’s arm). When giving speeches, he spoke at a lectern which he could grasp tightly to steady himself or holding the protective railing at the back of the last car of the presidential train. Due to widespread vaccination, we don’t see the effects of vaccine-preventable diseases and have forgotten the impacts these diseases have on people’s lives.  We return to Bill and Martin for a quick class discussion of whether the boys can be vaccinated at birth as well as during or after treatment. |
| Stefanie Joho’s story in the Washington Post | Tumor immunology | Stephanie Joho was dying of cancer as a result of Lynch Syndrome. We use an article the students read prior to class from the *Washington Post.* It discusses how her sister researched clinical trials, the roles of trials in drug development, the mechanism of action of the drug Keytruda and the ethical questions surrounding who is treated with such an expensive drug (McGinley 2017). |
| Bill and Martin, circling back to our case study protagonists one last time | Final exam | Students have the ability to submit potential exam questions for extra credit. This is representative of questions students have submitted concerning Bill and Martin:  Bill (our case study friend) is going into college. He needs to get a vaccination for bacterial meningitis (an intracellular bacteria). There are two options for vaccines: an inactivated, whole organism vaccine and a conjugate vaccine.  a. Of what is each vaccine composed?  b. List two other characteristics for each of the vaccines.  c. Which vaccine would be more appropriate for Bill? Why? |

**References for Supplemental Materials**

Geha R, Notarangelo L. Case Studies in Immunology: A Clinical Companion.

New York, NY: Garland Pub (2016).

McGinley L. ‘This is not the end’: Using immunotherapy and a genetic glitch

to give cancer patients hope. The Washington Post (2017, May 28). Available online at: <https://www.washingtonpost.com/national/health-science/this-is-not-the-end-experimental-therapy-that-targets-genes-gives-cancer-patients-hope/2017/05/28/cdce31de-365c-11e7-b373-418f6849a004_story.html>

Warren M. Second Patient Free of HIV After Stem-Cell Therapy. Nature

(2019). Available online at: https://www.nature.com/articles/d41586-019-

00798-3 (accessed July 17, 2019).
